# Four-dimensional flow cardiovascular magnetic resonance in tetralogy of Fallot: a systematic review

**DOI:** 10.1186/s12968-021-00745-0

**Published:** 2021-05-20

**Authors:** Ayah Elsayed, Kathleen Gilbert, Miriam Scadeng, Brett R. Cowan, Kuberan Pushparajah, Alistair A. Young

**Affiliations:** 1 Department of Anatomy and Medical Imaging, University of Auckland, Auckland, New Zealand; 2 Auckland Bioengineering Institute, University of Auckland, Auckland, New Zealand; 3 Institute of Environmental Science and Research, Auckland, New Zealand; 4 Department of Biomedical Engineering, King’s College London, London, UK

**Keywords:** Cardiovascular magnetic resonance, 4D flow, Tetralogy of Fallot

## Abstract

**Background:**

Patients with repaired Tetralogy of Fallot (rTOF) often develop cardiovascular dysfunction and require regular imaging to evaluate deterioration and time interventions such as pulmonary valve replacement. Four-dimensional flow cardiovascular magnetic resonance (4D flow CMR) enables detailed assessment of flow characteristics in all chambers and great vessels. We performed a systematic review of intra-cardiac 4D flow applications in rTOF patients, to examine clinical utility and highlight optimal methods for evaluating rTOF patients.

**Methods:**

A comprehensive literature search was undertaken in March 2020 on Google Scholar and Scopus. A modified version of the Critical Appraisal Skills Programme (CASP) tool was used to assess and score the applicability of each study. Important clinical outcomes were assessed including similarities and differences.

**Results:**

Of the 635 articles identified, 26 studies met eligibility for systematic review. None of these were below 59% applicability on the modified CASP score. Studies could be broadly classified into four groups: (i) pilot studies, (ii) development of new acquisition methods, (iii) validation and (vi) identification of novel flow features. Quantitative comparison with other modalities included 2D phase contrast CMR (13 studies) and echocardiography (4 studies). The 4D flow study applications included stroke volume (18/26;69%), regurgitant fraction (16/26;62%), relative branch pulmonary artery flow(4/26;15%), systolic peak velocity (9/26;35%), systemic/pulmonary total flow ratio (6/26;23%), end diastolic and end systolic volume (5/26;19%), kinetic energy (5/26;19%) and vorticity (2/26;8%).

**Conclusions:**

4D flow CMR shows potential in rTOF assessment, particularly in retrospective valve tracking for flow evaluation, velocity profiling, intra-cardiac kinetic energy quantification, and vortex visualization. Protocols should be targeted to pathology. Prospective, randomized, multi-centered studies are required to validate these new characteristics and establish their clinical use.

## Background

Tetralogy of Fallot (TOF) is a common serious form of congenital heart disease (CHD) and one of the first to be formally described historically [1–4]. The four main morphological features are (i) ventricular septal defect (VSD), (ii) right ventricular (RV) outflow tract obstruction, (iii) overriding aorta and iv) RV hypertrophy. Primary repair includes closure of the VSD, resection of infundibular muscle obstruction and relief of pulmonary stenosis. Additional repair of a stenosed pulmonary artery may be performed using a patch reconstruction. Residual anatomic and hemodynamic abnormalities in repaired TOF (rTOF) patients are highly prevalent, with pulmonary stenosis and pulmonary regurgitation (PR) being common. Chronic RV volume overload, akinesis or dyskinesis of the RV outflow tract wall, and a nearly universal right bundle branch block, trigger a sequence of pathophysiologic sequelae that lead to RV dilatation, and ultimately dysfunction and right sided heart failure [5, 6]. The long-term prognosis for TOF patients has improved in the past 80 years, however life expectancy is still lower in comparison with age-matched controls [2, 7]. A growing number of patients need continuous monitoring to determine whether further intervention, such as pulmonary valve replacement, is necessary [5, 7].

Longitudinal monitoring of cardiac output, RV volumes and pulmonic valve regurgitant fraction is typically performed using echocardiography and cardiovascular magnetic resonance (CMR). Although echocardiography is widely available and often used as a reference standard for blood flow velocity analysis [8], CMR has high signal-to-noise ratio and enables coverage of all anatomical regions [9–11]. Quantitative blood flow analysis using phase-contrast CMR of velocity (PC-CMR) enables precise quantification of velocity in the phase of each pixel of the CMR image. Four-dimensional PC-CMR (4D flow) enables evaluation of flow in multiple vessels as well as within the heart chambers in complex CHD [12–14].

Recently, a number of studies have reported 4D flow CMR in cardiac applications, including a systematic review [15] and a consensus statement [16]. We aimed to provide a systematic review of 4D flow applications in rTOF patients, to highlight clinical utility of 4D flow in rTOF, review validation data, compare acquisition parameters, and identify the differences and similarities between studies. Recommended sequences and techniques are highlighted for specific applications in rTOF patients, with the aim of encouraging future multi-center studies and more standardization in clinical protocols.

## Methods

### Search strategy

The Preferred Reporting Items for Systematic Reviews and Meta-Analysis (PRISMA) checklist was adhered to when structuring this article [17]. A comprehensive search was undertaken in March 2020 on Google Scholar and Scopus (this database enables a complete search of both MEDLINE and EMBASE). The search limitations included ‘Humans’ and ‘English Language’, with no time limitations. The search scope included the reference lists of included articles, citation tracking and manual reference searching.

The key words of the search were broken down to several searches to cover all the possible variations of the modality and the pathology (Tetralogy of Fallot), for example: (4D) OR (four-dimensional) AND (flow) OR [(3D) OR (three-dimensional)) AND ((cine) OR (time-resolved)) AND (PC) OR (phase contrast)] AND (CMR) (cardiovascular magnetic resonance) OR (MRI) OR (magnetic resonance imaging).

### Article screening and eligibility criteria

**Fig. 1:**
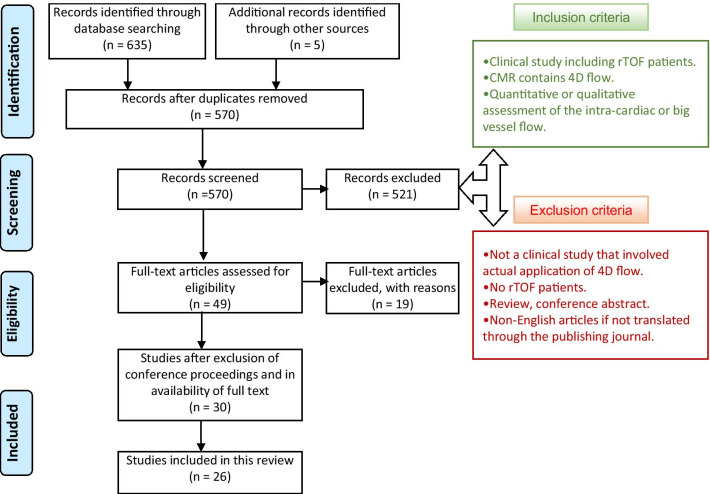
Flow diagram showing the stages of the systematic review process (adapted from Moher et al. [16])

The study selection process was performed by a single reviewer (AE) and reviewed independently (KG, MS) before a final review (AY, BC, KP). Disagreement in the articles were all resolved with the revision process. Once duplicates were removed, the titles and abstracts of the search results were assessed using a screening algorithm based on eligibility criteria as shown in Fig. 1. The studies that adhered to this screening had their full texts evaluated. Further exclusion was done based on the methodologies and study groups where these were not detailed in the abstracts.

### Data extraction

Characteristics of studies, authors, year, and participants were noted, if available. The study type and aim were determined, and classified into pilot, diagnostic and mechanistic studies. Study methodology details, including the study design, outcomes and conclusions, were evaluated in particular for the 4D flow protocol and analysis. A summary is presented in Table 1.

**Table 1:** Study list, analysis targets, applicability, validation methods, aims, study populations, main conclusions, and quantitative parameters

Study	Analysis	Applicability	Valid-ation	Aim	Number of cases, age range and sex		Conclusion	Quantitative parameters							
					CHD age (range) (n total, n male, n rTOF)	Normal control age (range) (n, n male)		Stroke volume^§^	Regurgitant fraction	Right and left pulmonary arteries	Systolic peak velocity	Q_S/_ Q_P_	EDV/ESV	Total kinetic energy	Vorticity
Nordmeyer et al. [11]	Venous & arterial flow pulmonary valve & AV flow	Highly clinically applicable	2D PC-CMR	2D vs nongated 4D vs gated 4D PC-CMR Venous and arterial Normal & CHD	19 ± 9 (n = 10, ♂8, 4 rTOF)	34 ± 7 (n = 7, ♂3)	4D flow CMR is accurate in arterial, venous, and pathological flow	●	●						
Van der Hulst et al. [26]	Pulmonary valve & tricuspid valve flow & RV diastolic function	Highly clinically applicable	2D PC-CMR & stroke volume	4D vs 2D PC-CMR Pulmonary valve and tricuspid valve flow, RV diastolic function Normal & rTOF	13 ± 3 (n = 25, ♂12, all rTOF)	14 ± 2 (n = 19, ♂12)	4D flow CMR is accurate for assessing pulmonary valve forward and backward flow in patients with rTOF and healthy children. Superior to 2D PC-CMR for tricuspid flow	●	●						
Geiger et al. [18]	Arterial flow and vortex visualisation	Potentially clinically applicable	None	Feasibility of Vortex flow visualisation and retrospective flow quantification by 4D CMR Normal & rTOF	12 ± 8 (2–24) (n = 10, ♂5, all rTOF)	26 ± 1(25–27) (n = 4, ♂NS)	4D flow CMR analysis may provide valuable data on both intracardiac and pulmonary vascular flow		●	●	●				●
Hsiao et al. [10]	Systemic & pulmonary flow	Highly clinically applicable	2D PC-CMR	2D PC CMR vs 4D CMR CHD patients	(3–29) (n = 18, ♂NS, 9 rTOF)	–	4D flow CMR has higher consistency than 2D PC-CMR					●			
Hsiao et al. [42]	Valves and shunts	Highly clinically applicable	Echo	The potential of PICS 4D flow and specialized imaging software in valvular insufficiency and intracardiac shunts CHD patients	(1–21) (n = 34, ♂19, 5 rTOF)	–	PICS 4D flow CMR is sufficient in identification of intracardiac shunting and haemodynamically significant valve regurgitation		●			●			
Hsiao et al. [9]	Ventricular volumes and flow	Potentially clinically applicable	2D PC-CMR & stroke volume	Accelerated 4D PC CMR vs 2D PC CMR flow and volume measurements CHD patients	(1–29) (n = 29, ♂NS, NS rTOF)	–	PICS 4D flow CMR is accurate for ventricular volumetry and flow	●				●	●		
François et al. [19]	Arterial pulmonary flow and vortex	Potentially clinically applicable	None	Feasibility of 4D flow (VIPR) in flow visualisation and retrospective flow quantification by 4D CMR Normal & rTOF	20 ± 12 (7–43) (n = 11, ♂5, all rTOF)	34 ± 13 (21–54) (n = 10, ♂6)	VIPR 4D flow CMR is feasible. Analysis of types of TOF repair using 4D flow will be necessary in outcome prediction	●	●		●				●
Tariq et al. [20]	Venous & arterial flow quantification pulmonary valve & AV flow	Potentially clinically applicable	None	Evaluate the precision and accuracy of PICS 4D venous and arterial flow quantification CHD patients	3.0–12.0 (n = 22, ♂5, 8 rTOF)	–	PICS 4D flow CMR is accurate for venous flow quantification	●				●			
Nordmeyer et al. [27]	Arterial flow and valves	Highly clinically applicable	2D PC-CMR & Echo	4D flow vs 2D PC-CMR Valve stenosis and flow Normal, valve stenosis	26 ± 10 (n = 18, ♂9, 4 rTOF)	34 ± 7 (n = 7,♂3)	4D flow CMR improves quantification accuracy of peak flow velocities in semilunar valve stenosis	●			●				
Giese et al. [22]	Arterial and pulmonary flows, volumes and vortex	Potentially clinically applicable	2D CMR	k-t PCA 4D flow vs 2D PC-CMR flow and volume measurements Normal, CHD patients	1–21 (n = 9, ♂NS, 1 rTOF)	23–40 (n = 10, ♂NS)	k-t PCA 4D flow CMR is feasibile in CHD	●			●	●			
Jeong et al. [36]	Ventricular kinetic energy	Highly clinically applicable	None	Ventricular kinetic energy with 4D flow Normal and rTOF	20 ± 12 (7–43) (n = 10, ♂5, all rTOF)	39 ± 15 (n = 9, ♂6)	VIPR 4D flow CMR kinetic energy is a novel non-invasive method of monitoring cardiac efficiency	●						●	
Hsiao et al. [28]	Valvular flow	Highly clinically applicable	2D CMR	Feasibility of PICS 4D flow for RF and valve flow CHD patients	1–15 (n = 34, ♂19, 8 rTOF)	–	PICS 4D flow CMR with valve tracking is accurate for valvular flow and RF	●	●				●		
Gabbour et al. [21]	Arterial Peak velocities and stroke volumes	Potentially clinically applicable	2D PC-CMR & Echo	4D vs 2D PC-CMR and Echo Aortic and pulmonary flow and volume measurements CHD patients	13 ± 6 (4–29) (n = 50, 10 rTOF)	–	4D flow CMR is a clinical alternative to 2D PC-CMR in children and young adults	●	●	●	●				
Hirtlir et al. [12]	Arterial and pulmonary flows, volumes and vorticity	Highly clinically applicable	None	Analysis of flow and vorticity in the right heart by 4D flow Normal & rTOF	12 ± 6 (1–24) (n = 24, ♂16, all rTOF)	23 ± 2 (21–26) (n = 12, ♂7)	Quantitative intracardiac vorticity in rTOF patients can be correlated with flow and volumes	●	●						●
Hanneman et al. [29]	Ventricular volumes and flow	Highly clinically applicable	bSSFP	4D flow with ferumoxytol for volume and mass CHD patients	6 ± 5 (1–11) (n = 22, ♂10, 8 rTOF)	–	4D flow CMR with ferumoxytol enables accurate evaluation of mass and volume as well as flow in a single acquisition						●		
Chelu et al. [30]	Pulmonary flow	Highly clinically applicable	2D PC-CMR	Evaluate a cloud-based platform for 4D flow analysis Quantify forward flow, regurgitation, and peak systolic velocity over the pulmonary artery CHD patients	38 ± 15 (n = 52, ♂25, 4 rTOF)	–	Bulk flow and pulmonary regurgitation can be accurately quantified using 4D flow CMR analysed with a cloud based application	●	●		●				
Hussaini et al. [37]	Ventricular kinetic energy	Highly clinically applicable	None	Time-resolved versus time-averaged ventricular segmentation on 4D CMR kinetic energy calculations CHD patients	24 ± 21 (8–61) (n = 5, ♂2, 3 rTOF)	27 ± 2 (24–31) (n = 10, ♂6)	Time averaged segmentation is more efficient but overestimates time resolved values						●	●	
Fredriksson et al. [31]	Ventricular kinetic energy	Highly clinically applicable	None	RV turbulent kinetic energy & relationship with RV remodelling Normal & rTOF	21–65 (n = 17,♂9, all rTOF)	31 ± 11 (22–54) (n = 10,♂NS)	Total KE in the RV of patients with rTOF increases with RV volumes and regurgitant fraction		●					●	
Driessen et al. [23]	Tricuspid valvular flow	Highly clinically applicable	2D PC-CMR & echo	2D vs 4D flow vs Echo for tricuspid valve flow and regurgitation CHD, pulmonary hypertension & normal	43 ± 17 (n = 67, ♂35, ~ 21 rTOF)	41 ± 11 (n = 21,♂14)	4D flow CMR shows good agreement to 2D PC-CMR. 38% had different grading to echo	●	●						
Sjoberg et al. [33]	Ventricular kinetic energy	Highly clinically applicable	None	RV and LV kinetic energy Normal & rTOF	29 ± 12 (n = 15, ♂10, all rTOF)	30 ± 7 (n = 14, ♂12)	RV Total KE higher in rTOF and highest in restrictive physiology	●	●					●	
Sjoberg et al. [32]	Haemodynamic forces	Highly clinically applicable	None	Ventricular haemodynamic forces Normal & rTOF	29 ± 13 (n = 18, ♂11, all rTOF)	31 ± 7 (n = 15, ♂10)	Differences in forces versus control subjects remain after pulmonary valve replacement	●	●						
Robinson et al. [34]	Ventricular kinetic energy	Highly clinically applicable	None	RV turbulent kinetic energy relationship with RV remodelling Normal & rTOF	14 ± 8 (n = 21, ♂8, all rTOF)	16 ± 3 (n = 24, ♂11)	Total KE in the RV of patients with rTOF increases with RV volume and regurgitant fraction	●	●					●	
Lee et al. [58]	Aortic flow	Highly clinically applicable	None	Flow in the ascending aorta and relationship with aortic dilatation Normal & rTOF	29 ± 8 (n = 44, ♂25, all rTOF)	34 ± 9 (n = 11, ♂10)	Aortic dilatation, wall shear stress and flow jet angle changed in rTOF patients				●				
Isorni et al. [25]	Pulmonary and aortic flow	Highly clinically applicable	2D PC-CMR	Pulmonary and aortic flow Normal & rTOF	18 ± 10 (2–54)^#^ (n = 50, ♂NS, all rTOF)	NS (n = 10, ♂NS)	4D flow CMR is feasible and more consistent pulmonary vs aortic flow in rTOF	●	●	●	●				
Jacobs et al. [24]	Ventricular volumes and flow	Highly clinically applicable	2D PC-CMR	2D vs 4D CMR rTOF	16 ± 4 (n = 34, ♂14, 31 rTOF)	–	4D flow CMR with gadobenate dimeglumine accurate for flow and volumes	●	●	●		●	●		
Schafer et al. [35]	Aortic flow and vorticity	Highly clinically applicable		Aortic flow and LV vorticity Normal & rTOF	11 ± 3 (n = 14, ♂9, all rTOF)	10 ± 2 (n = 10, ♂6)					●				●

*CHD* congenital heart disease, *rTOF* repaired tetralogy of Fallot, *Qs/Qp* systemic/pulmonary total flow ratio, *EDV* end-diastolic volume, *ESV* end-systolic volume, *KE* kinetic energy, *PC-CMR* phase contrast cardiovascular magnetic resonance, *LV* left ventricle, *RV* right ventricle

### Quality assessment

The quality of the included studies was assessed by AE using a modified Critical Appraisal Skills Programme (CASP) tool, provided in Table 2. The questions were modified and set criteria designed for the purpose of estimation of the applicability of the studies selected, rather than criticism of the proposed methods. The presence or absence of criteria are not relevant to the strength of the presented work but to the completeness for reapplication of the same methodologies to similar study groups.

**Table 2:** 4D CMR sequence parameters

Study	Field (Tesla)	Scanner type	Acc type	Acceleration factor		Sampling resolution (mm)		Temporal resolution (ms)	Flip angle	No. of phases	VENC (cm/s)	Scan time (min)	Contrast	Respiratory gating	Cardiac gating
				In-Plane	Slice	In-Plane	Slice		(°)	(n)					
Nordmeyer et al. [11]	3	Phiips Achieva	No	No	No	2.5–2.6	2.5	NS	5	24	150–400	8.6 ± 1.2	No	Yes and No	Retro
Van der Hulst et al. [26]	1.5	Philips Intera	EPI	5	No	2.9–3.8	4	NS	10	30	150	NS	No	No	Retro
Geiger et al. [18]	1.5/3	Siemens Avanto or Trio	GRAPPA	2	No	1.7–3.2	1.6– 2.5	37.6–40.8	7–15	NS	150–200	10–20	No	Yes (ADNG)	Pro
Hsiao et al. [10]	1.5	GE TwinSpeed	GRAPPA	2	No	0.9–2.3	3–5	33–42	15	NS	150–400	9–21	Gd	No (*EXORCIST)*	NS
Hsiao et al. [42]	1.5	GE TwinSpeed	PICS	1.4–2.2	1.4–2.2	0.8–1.8	1.2–3.4	31–86	15	NS	120–350	5–15	Gd	No (*EXORCIST)*	NS
Hsiao et al. [9]	1.5	GE TwinSpeed	PI	2	No	1.2–2.3	3–4	29–64	15	NS	150–500	4–14	Gd	No	NS
Hsiao et al. [9]^*^	1.5	GE TwinSpeed	PICS	1.6–2.2	1.6–2.2	0.8–1.7	2–3.4	33–86	15	NS	150–300	7–15	Gd	No	NS
François et al. [19]	1.5/3	GE HDx or MR750	PC VIPR	NS	NS	1.0–1.3	1.02–1.25	25–44	7–20	NS	40–400	9–17	Gd in CHD only	Yes (ADNG)	Retro
Tariq et al. [20]	1.5	GE TwinSpeed	PICS	2.0–2.2	2.0–2.2	NS	NS	53	15	NS	150–350	7–15	Gd	No (*EXORCIST)*	NS
Nordmeyer et al. [27]	1.5/3	Philips Achieva	SENSE	2	NS	2.5	2.5	NS	5	24	150–500	7–20	No	No	Retro
Giese et al. [22]	1.5	Philips Achieva	k-t SENSE/k-t PCA	2–12		1.2–2.5/1.4–2.5	1.7–2.5	24–32	6	24	130–400	4–8	No	Yes	Retro
Jeong et al. [36]	1.5/3	GE HDx or MR750	PC VIPR	NS	NS	1.3	NS	35–44/25–27	NS	NS	40–400	9–17	Gd	Yes	Retro
Hsiao et al. [28]	1.5	GE TwinSpeed	PICS	1.6–2.2	1.6–2.2	0.8–1.9	2–3.4	33–86	15	20	150–300	7–5	Gd	No (*EXORCIST)*	NS
Gabbour et al. [21]	1.5	Siemens Avanto or Aera	GRAPPA/k-t GRAPPA	2/5	NS	2.7–4.1	2.0–3.5	37–40	15	9–24	100–250	13 ± 5	Gd	Yes (ADNG)	Pro
Hussaini et al. [37]	3	GE MR750	PC VIPR	NS	NS	1.32	1.32	NS	8	20	150	NS	No	Yes	Retro
Hirtlir et al. [12]	1.5/3	Siemens Avanto or Trio	No	No	No	1.6–3.2	2.4–3.6	38–41	15	NS	150–200	10–20	Gd in CHD only	Yes (ADNG)	Pro
Hanneman et al. [29]	3	GE MR750	PICS	2.4	4.4	0.8	1.4	73–226	15	-	150–300	5–13	Ferumoxytol	No	Retro
Chelu et al. [30]	1.5/3	GE MR450 or MR750	PICS	2	2	1.8–2.1	2.8	60	15	-	250	8–10	Gd	No	Semi-Retro
Fredriksson et al. [31]	1.5	Philips Achieva	SENSE	2	No	2.8–3	2.8–3	48–49	8	NS	100–120	15–20	No	Yes (ADNG)	Retro
Driessen et al. [23]	1.5	Philips Ingenia	EPI	5	No	3.4–3.7	3.5	NS	10°	30	150	3.5–7	No	No	Retro
Sjoberg et al. [33]	1.5	Philips Achieva or Siemens Aera	SENSE	2	No	3	3	45	8	40	100	NS	No	NS	Retro
Sjoberg et al. [32]	1.5	Philips Achieva or Siemens Aera	SENSE	2	2	3	3	45	8	40	100	NS	Gd in CHD only	No	Retro
Robinson et al. [34]	1.5	Siemens Avanto or Aera	NS	NS	NS	1.4–4.5	1.4–3.5	36–45	15	NS	100–250	NS	Gd	NS	NS
Lee et al. [58]	3	GE Trio	NS	NS	NS	2.1–1.6	3	41–60	15	NS	200	NS	NS	Yes (ADNG)	NS
Isorni et al. [25]	1.5	GE Discovery MR450	NS	NS	NS	2.1	2.4	NS	10	30	200–400	NS	Gd	No	Retro
Jacobs et al. [24]	1.5	GE Optima 450 W	PICS	2.4	4.4	0.8	1.4	45–73	15	20	250	NS	Gd	No	Retro
Schafer et al. [35]	3	Philips Ingenia	NS	NS	NS	2	2–2.8	38–48	10	14–16	150	10–12	No	Yes (ADNG)	Retro

*Acc* acceleration, *EPI* echo planar imaging, *PI* parallel imaging, *PICS* parallel imaging compressed sensing, *VIPR* isotropic-voxel radial projection imaging, *SENSE* Sensitivity encoding, *GRAPPA* generalized autocalibrating partial parallel acquisition, *PCA* principal component analysis, *NS* Not specified, *ADNG* adaptive diaphragm navigator gating. *Pro* prospective, *Retro* retrospective, *Gd* Gadolinium, *CHD* congenital heart disease. *EXORCIST* Respiratory compensation with k-space phase reordering. Scanner manufacturers: Achieva, Intera, Ingenia: Philips Healthcare, Best, Netherlands; Avanto, Trio, Aera: Siemens Healthineers, Erlangen, Germany; Twinspeed, HDx, Discovery MR450, MR750, Optima 450 W: General Electric Healthcare, Milwaukee, Wisconsin, USA. *Two protocols employed in this paper

Answers of ‘yes’ scored 1 point, whereas answers of ‘no’ or ‘indeterminate’ scored 0 points. Total scores were converted to percentages and studies were allocated to one of three categories; ‘highly clinical applicable’ for a score of 67–100%, ‘potentially clinically applicable’ for 34–66% and ‘less clinically applicable’ for 0–33%.

### Quantitative assessment

A generalized meta-analysis was not possible for this systematic review as much of the research is exploratory and preliminary, with considerable heterogeneity in the study outcomes. However, analysis of the similarities and differences was performed. A narrative review is provided.

## Results

### Search strategy

A search of the electronic databases produced 635 articles. After removing duplication, 570 articles remained. Based on the eligibility criteria, title and/or abstract were initially screened, and 30 articles remained which met the initial search criteria as potentially relevant to the current study. After reading the full-text articles, 4 articles were excluded. Therefore, the final selection of studies included 26 articles. A summary of the process is presented in Fig. 1.

### Description of the included studies

Twenty-six studies were included in this systematic review, the details are described in Table 1. None were below 59% applicability thus no studies had low applicability. Of the 26 studies, 6 scored above 80%, 6 studies scored 70–80% and the rest were between 59 and 70%. Studies that had been performed more recently yielded higher scores, reflecting a more targeted application of the technique.

### Aim of the studies

Studies could be broadly classified into:

i) Pilot studies, qualitative evaluations [18, 19] and preliminary quantification studies.
ii) Validation of new methods (mainly acceleration techniques) of 4D flow acquisition [9, 20, 21], for example comparing under-sampled data against fully sampled data [22].
iii) Validation against 2D CMR along with qualitative and quantitative description of blood flow [9–11, 21–30].
iv) Identification of novel flow features such as vortices in the right side of the heart in rTOF patients compared with normal populations [12, 19] and the effects of different surgeries on vortices in the heart [18] as early prognostic factors in ventricular changes and valvular malfunction.

### Imaging validation methods

Fourteen of 26 (54%) studies had a quantitative comparison with other modalities. All these studies concluded that 4D flow results demonstrated a high agreement via several statistical tests and no significant differences in flow except in a few cases, with rational explanations for these results. Four studies involved comparison with echocardiography. Echocardiography has high availability and good temporal resolution; however, flow quantification is limited by the geometry and angulation of the transducer as well as the availability of acoustic windows [8]. In a study that focused on the tricuspid valve, 38.5% of patients were classified differently by at least one grade using quantitative 4D flow CMR when compared to the echocardiographic assessment [23].

Several studies compared 4D flow with 2D PC-CMR, since 2D acquisition is historically well validated and standardized [9–11]. However, 2D slices need to be accurately localized and the results are often inconsistent (e.g. the flows in the left and right pulmonary branches may not add up to the flow in the main pulmonary artery) due to separate breath-hold acquisitions. An alternative comparison can be made between stroke volume integrated from ventricular outflow and volumetric stroke volume calculated from anatomical cine balanced steady state free precession (bSSFP) planimetry.

### Study populations

**Fig. 2:**
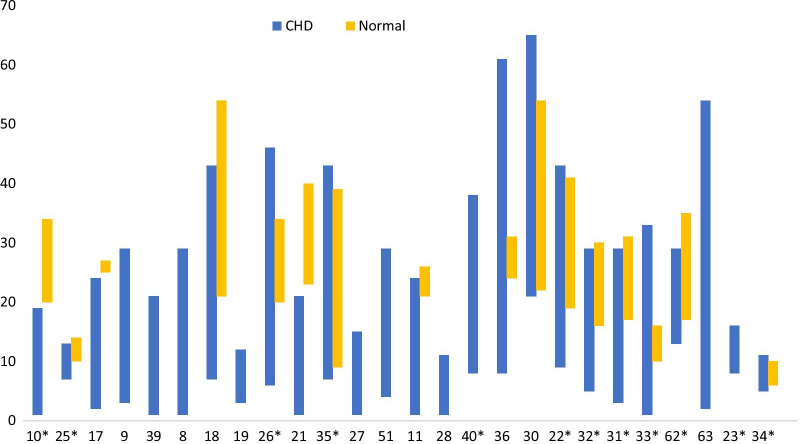
Participants age demographic profile. * estimated from mean ± 2*s.d

Several studies compared the patient population against a normal population that had no known history of cardiovascular disease. In earlier studies there was a significant difference in age between the groups (Fig. 2), with older volunteers. However later studies had more age-matched study populations [26, 31–35] (Fig. 2).

### Quantified parameters

**Fig. 3:**
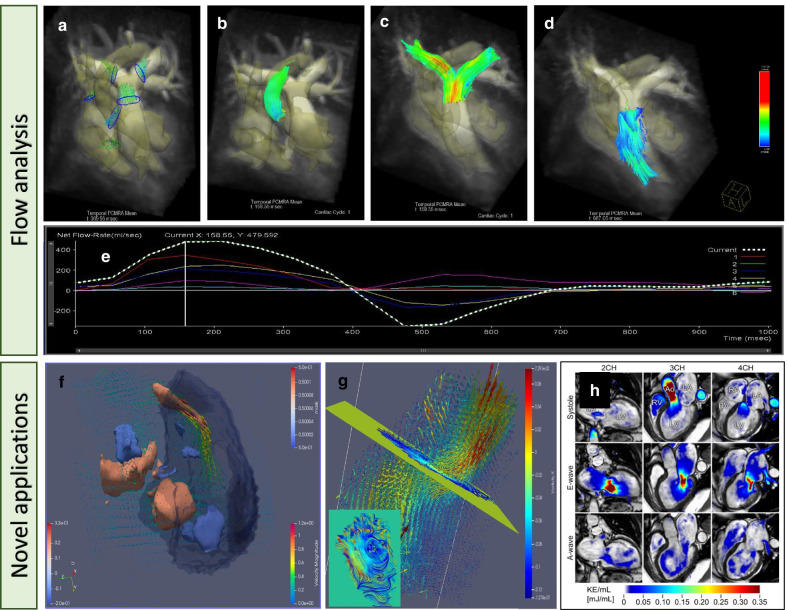
4D flow analysis applications. (**A**) manually placed seed positions; (**B**) particle streamline visualization of the ascending aorta; (**C**) main pulmonary and branches forward flow; (**D**) main pulmonary artery regurgitation; (**E**) flow analysis: Net flow across cardiac cycle; (**F**) 3D vortex core extraction in the right ventricle; (**G**) 2D visualization of vortices in the right ventricle; (**H**) kinetic energy mapping from Kanski et al. JCMR (2015) 17:111 DOI 10.1186/s12968-015-0211-4 used under the terms of the Creative Commons Attribution 4.0 International License (http://creativecommons.org/licenses/by/4.0/)

Quantified parameters included arterial or venous flow such as systolic peak velocities, or stroke volume from net flow across outflow valves, or evaluation of the valvular efficiency through regurgitant fraction (Table 1). Qualitative analysis of the flow and vorticity in different areas of the heart and great vessels were also evaluated [12, 18, 19, 35]. In addition to the measurements of basic flow volumes and velocities, the estimation of derived hemodynamic biomarkers such as wall shear forces and a resistance index [19] and ventricular kinetic energy measurements [31, 36, 37] have been proposed. Ventricular kinetic energy (KE) and its applications have been more recently investigated [33, 34, 38]. In some studies, end-diastolic (EDV) and end-systolic volumes (ESV) were quantified from the 4D flow data. The following is a summary of the main findings with respect to these parameters: In total there were 18/26 (69%) studies on stroke volume, 16/26 (62%) on regurgitant fraction, 4/26 (15%) on right versus left pulmonary artery flow, 9/26 (35%) on systolic peak velocity, 6/26 (23%) on Qp:Qs, 5/26 (19%) on EDV and ESV, 5/26 (19%) on KE, and 4/26 (15.3%) on vorticity. Commonly examined flow parameters are illustrated in Fig. 3.

### Stroke volume

Stroke volume was quantified as volume of effective antegrade flow per heartbeat, i.e. the difference between forward and backward flow volume above the aortic or pulmonary valve [11, 13] in the absence of regurgitant volumes. Good agreement between 2 and 4D was found in several studies. The average mean of differences represented < 1% of the overall mean value for arterial and < 3% for venous stroke volume [9, 11, 26, 27]. Other studies found good internal consistency through indirect measurement of cardiac output using stroke volume from cine slice planimetry. Nevertheless, some studies reported some significant bias, for example Giese et al [21]. reported an underestimation of stroke volume by 2.5 ± 8.4 ml with 4D flow corresponding to 5.6 ± 14.9% with respect to the stroke volumes derived from the 2D flow datasets.

Internal validation of stroke volumes at different levels of the same vessel at different levels in the ascending aorta and pulmonary trunk did not show significant differences [27]. However, there was a significantly higher variation in patients compared to healthy subjects, which may be due to patients having complex flow patterns leading to signal loss in the presence of some turbulent flows [24].

No statistically significant difference was observed between 4D flow (magnitude images) and bSSFP measurements of EDV, ESV and stroke volume in some studies using contrast [8, 23, 27, 36]. However, Hanneman et al. [28] observed that RVEDV and ESV were underestimated by 4D flow compared to bSSFP and attributed to differences in basal slice selection. Conversely, they found no significant difference in left ventricular (LV) or RV mass quantification with 4D flow compared to bSSFP acquisitions in the same study.

### Regurgitation

Regurgitant fraction is the diastolic reversed flow expressed as a percentage of forward flow or the percentage of backward flow volume into the corresponding ventricle during diastole [13, 39]. Regurgitant fraction is an important parameter in rTOF patients because of common pulmonary valve insufficiency [25]. Due to the presence of vortices in the pulmonary artery, antegrade and retrograde flows can occur simultaneously in the same cardiac phase. Therefore, PR fraction computed using pixel-wise integration can be greater than that calculated by average velocity integration [40]. PR volume is also important and may reflect the severity of regurgitation better than regurgitant fraction [41].

Several studies reported no significant differences between 2 and 4D flow CMR for both antegrade and retrograde flow [11, 21, 25]. However, van der Hulst et al. [26] observed significant differences in pulmonary valve backward flow volumes between 2D PC-CMR and 4D flow but no significant differences between the 4D flow and planimetric estimates (RV stroke volume minus LV stroke volume). On the other hand, a significant difference was seen between patient and normal populations [19]. Chelu et al. reported that 4D flow had a sensitivity of 83% (95%CI: 36–100%), a specificity of 98% (95% CI: 88–100%), and an accuracy of 96% to identify patients with RF > 20% [30]. Four studies observed ejection fractions concluding good correlations between 2D flow and 4D flow [12, 29, 42, 43].

The tricuspid valve is also of importance when assessing right sided CHDs. Tricuspid regurgitation (TR) is independently associated with both increased morbidity and increased mortality in CHD patients [23]. The degree of TR is an important diagnostic parameter in the decision making of valve replacement in rTOF patients [23]. The tricuspid valve has range of motion which is increased in the setting of severe TR [44] and quantification of the regurgitation fraction is difficult due to the increased in-plane motion of the valve annulus. TR volume can also be derived from the difference in planimetric RV stroke volume and direct flow measurement across the pulmonic valve [23].

Altering geometry and valve structure changes flow patterns, increase the difficulty of TR assessment. Some studies have validated tricuspid flow measurement by 4D flow in patients without TR [26] and patients with TR and complex RV geometry [23, 26]. Both studies demonstrated that quantifying tricuspid valve flow using retrospective valve tracking was possible in 100% of the imaged patients. Effective flow was reported to be significantly overestimated by 2D flow compared to both other methods, and regurgitant fraction was significantly underestimated by 2D flow in both studies. In one study 39% of TR patients were classified differently by at least 1 grade using 4D flow compared to echocardiography [23]. Strong intra /inter-observer agreement was also demonstrated with 4D flow ICC > 0.91. Earlier studies had reported an agreement with an ICC = 0.93 to 0.94 [45, 46]. The difference between 4D flow and 2D PC-CMR flow was also attributed to motion of the valve annulus through the plane of the image with 2D, and the breathing artifact if 4D was obtained during free breathing, and finally interscan variability was also considered [23, 26].

### Flow and peak velocity

Aortic and pulmonic flow rates showed good agreement between 4 and 2D flow using Bland–Altman evaluation in most of the studies. One study compared several 4D flow segmentation approaches with 2D, showing differences in flow rate of 1–2% and 0.3–0.4 L/min or 6–8% in cardiac output [10]. The results of this study group are similar to those of a study on healthy subjects, where estimated stroke volumes were on average 3% greater with 4D flow than with 2D PC-CMR [47]. Nevertheless, a 12% difference with a higher 2D estimate was also observed [9]. Chelu et al. [43] reported that 4D flow underestimated peak systolic velocities, whereas Nordmeyer et al. [27] reported the opposite, with 10% higher peak velocities with 4D flow compared to 2D PC-CMR at the level of highest velocity. The same studies also noted better correlation with echocardiography with 4D flow than 2D PC-CMR. This was also observed by Gabbour et al. [21] who found that 2D PC-MR significantly underestimated aortic and main pulmonary artery peak systolic velocities compared to volumetric 4D flow and echo. Their study suggested that these findings were due to the 4D flow assessment of the entire vessel volume.

### Right atrium

It has been suggested that the conversion of rotational flow into helical flow in the right atrium may be a method of conserving atrial KE during RV filling [48]. This is supported by studies that observed a clockwise right atrium vortex in all their healthy subjects [12, 19]. A right handed helical flow was also observed between the right atrium and the RV during tricuspid valve diastolic filling, which was suggested to have an influence on the KE used by the atrium [31, 36], also described as an inflow jet with an adjacent vortex formation [12, 49]. rTOF patients showed abnormal timing of right atrium filling, more in diastole than in systole [19] with additional anticlockwise vortices [12].

### Right ventricle

Following the vortex observed in the tricuspid valve, parts of the intraventricular flow were instantaneously directed towards the RV outflow tract during diastole to be followed by slight helical flow in the RV outflow tract during systole [12]. Vortices and changes in flow were minimal at the apex. In rTOF patients flow features were very heterogeneous in direction, with large PR jets also directed toward the RV apex, and increased number of vortices [12, 19]^.^

### The pulmonary artery and its branches (right and left pulmonary arteries)

Whereas flow in healthy subjects is relatively uniform, rTOF patients exhibit asymmetric flow distribution in the pulmonary arteries, severe regurgitation and pronounced vortices [19] consisting of increased helical and vertical flow patterns. High peak velocities > 1.5 m/s have been observed in patient populations but not in normal control groups [18, 19].

### Superior/inferior vena cava

Two studies observed flow in the venae cavae. The flow from the superior vena cava and inferior vena cava into the right atrium was considered normal when greater flow occurred during systole than diastole. This occurred in almost all normal subjects (although it was reversed in 10%, attributed to intrathoracic and intraperitoneal pressure fluctuations) and ~ 20% of subjects with rTOF [18, 19].

### Pulse sequence parameters

Table 2 shows a comparison of sequence parameters between the studies included in this review. Some parameters were very similar between studies whereas others were adjusted to optimize the visualization of the structure of interest. The following sections summarize the main differences. Of note is that 16 studies used contrast to provide enhanced signal-to-noise and higher acceleration [28]. Most of these studies used gadolinium, although 2 used ferumoxytol (Table 2).

### Magnet strength

The types of CMRI units were variable with 16 studies using 1.5 T, 5 studies using 3 T, and 6 studies incorporating both field strengths. No studies compared 1.5 vs 3 T in accuracy or visualization of flow parameters.

### The acceleration type and factor and scan duration

The clinical applicability of 4D flow is aided by shortening the scan duration [13]. Improvements of reconstruction algorithms dedicated to phase-contrast imaging enable acceleration factors greater than 5. Parallel imaging acceleration was common with the shortest scan duration being 3.5 min [23]. Parallel imaging compressed sensing (PICS), echo planar imaging (EPI) and isotropic voxel radial projection imaging (VIPR) were often used in additional to conventional parallel imaging methods (SENSE and GRAPPA). The average acceleration factor was 2 (in-plane acceleration factor). The average scan time across studies was 11 min, although this was highly dependent on heart rate.

### Respiratory gating

Use of respiratory gating with either navigator or bellows was mixed, with 14 studies not using respiratory gating. Several studies examined non-gated 4D flow CMR against other methods, suggesting good agreement with conventional breath-hold 2D and respiratory gated 4D flow CMR methods [11, 50]. Respiratory compensation with k-space phase reordering (EXORCIST, General Electric Healthcare, Milwaukee, Wisconsin, USA), was used in some studies [9, 10]. Adaptive diaphragm navigator gating was used by others although the acceptance window was typically not reported (with the exception of 3–5 mm in [22]), and several studies used respiratory bellows [19, 37].

### Cardiac gating

Most studies used retrospective cardiac gating in the acquisition. Nevertheless, three studies conducted prospective gating [8, 12, 18], and vector electrocardiogram (ECG) gating was used by several studies [9, 10, 20, 28]. One study implemented a semi-retrospective gating [30].

### Velocity encoding (VENC)

VENC is defined as the (positive or negative) maximum velocity that can be detected without phase wrap. Prior approximate knowledge of peak velocity expected in the vessel of interest is thus essential. If the VENC is too low, either the scan must be repeated or antialiasing correction needs to be performed. Decreasing VENC corresponds to increasing the strengths and duration of the velocity encoding gradients, leading to longer echo and repetition times [16, 51]. However, VENC should be set as low as possible to achieve optimal phase signal-to-noise ratio. Most studies used 150 cm/s (range 40–500). Lower VENC is appropriate for intraventricular vortices [19] or KE [52] quantification. The highest VENC was 500 cm/s which may be necessary for peak systolic velocities in jets [27, 42]. Anti-aliasing pre-processing was performed by some studies to ameliorate this issue [21]; however, post processing antialiasing algorithms are becoming more common.

## Discussion

### Clinical application of 4D flow in rTOF

This systematic review emphasized the applicability of the 4D flow CMR in rTOF clinical evaluations. Velocity and flow volume measurements are required for evaluating severity of disease, and have been validated against 2D PC-CMR [8, 10, 11, 23–27, 30, 53] and echocardiography [8, 23, 27, 28]. Visualization of anatomy and classification of pulmonary blood supply is necessary to plan appropriate surgical management in many forms of CHD [54]. Applicability was highly promising, especially where studies addressed the diagnostic needs of more complex cases requiring quantitative flow measurements on both the venous and arterial sides [10, 27]. Accuracy was internally validated through employing the ‘conservation of mass’ principle, comparing volumes that are expected to be equal in absence of valvular malfunction or shunts [16]. This was done by comparison of input venous return and output arterial volumes [20], the comparison between the sum of branches to the main pulmonary artery [25], as well as the systemic versus the pulmonary circulation [10, 22]. Superiority of 4D flow CMR in rTOF was evident through higher correlations and lower mean differences than 2D PC-CMR. This is in agreement with studies in other patient groups [55, 56], which also found greater inconsistency in 2D PC-CMR, likely due to changing output during different breath-holds [25, 57].

Visualization of the pulmonary arteries is generally difficult to evaluate using echocardiography, due to acoustic-window limitations, but was feasible and recommended by 4D flow [18]. Further, mean velocities and net flow calculations with echo is dependent on flow profile and vessel cross-sectional area, which may be less accurate with complex arterial flow and distorted anatomy [16]. Complex flow patterns are particularly relevant in patients with dilated main pulmonary arteries, as the accuracy of flow measurements is compromised in the presence of focal turbulence, through-plane motion of stenotic semilunar valves, or the postoperative distorted anatomy itself [57].

Quantification of PR holds particular importance in the timing of pulmonary valve replacement [5]. 4D flow is recommended over multiple 2D PC-CMR acquisitions for evaluating residual pulmonary stenosis, branch artery stenosis, and PR in candidates for pulmonary valve replacement [12, 18].

Retrospective valve tracking for valvular flow quantification using 4D flow CMR had high clinical applicability in studies that covered the whole heart to investigate several pathologies [15]. For example, flow measurements have been recommended to be performed not only at the pulmonary but also at the aortic valve [28, 58] in the rTOF group. Early TOF repair may cause a decrease in aortic compliance and increase in wall shear stress which would increase the risk of late aortic complications in the rTOF adult [35]. Tricuspid valve [23] flow visualization and anatomical details were also found to be essential to improve preoperative selection of the type of procedure, for example patients that would benefit from tricuspid valve annuloplasty [59, 60]. 4D flow has better spatial coverage in all dimensions enabling visualization of flow jets through stenotic valves [27]. However, in children, high steady-state heart rate and respiration frequency [27] potentially influence flow measurement accuracy [26].

Interobserver agreement were moderate to high in 4D flow and bSSFP estimates of ventricular volume, ejection fraction, and mass, with no significant differences in agreement between techniques found. However, agreement with 4D flow CMR was higher for the LV than the RV, likely due to the complexity of RV especially in CHD patients [12, 29].

4D flow can reduce the total time of CMR, with an average scan time across studies of 12 min (range from 4 to 20 according to the acceleration type). This has reduced the duration of anesthesia required for younger pediatric patients [9] or patients with decompensated heart failure [16].

### Novel quantitative and qualitative parameters

4D flow enables greater understanding of pathophysiology and several studies in this systematic review have examined new diagnostic and prognostic flow parameters [16]. These augment conventional measures of velocities and regurgitant fractions with new measures of flow characteristics and energetics, which could aid prediction of future outcomes. Qualitative assessment of blood flow patterns is a distinctive feature of 4D flow and further study is required to relate these measures with outcomes [12, 18]. Studies have covered a wide range of pathology, from rTOF pediatric and adult cohorts to adult and pediatric healthy subjects. Vortex flow has been corelated with pulmonary hypertension [61], and turbulent flow may contribute to the development of RV remodeling, RV outflow tract fibrosis, and other complications seen in patients with rTOF [19]. Furthermore, surgical outcomes may be additionally affected by postoperative vessel dilation due to vortices, requiring standardization and automated quantification [62]. Flow vortices may provide independent prediction of surgical outcome [18, 19, 63, 64] and further research on automated vortex extraction is required [15, 65].

Diastolic RV function and biventricular hemodynamic forces may be a useful prognostic tool [32]. A restrictive right ventricular filling pattern has been correlated postoperatively with slower recovery after repair [66]. With 4D flow CMR, summation of pulmonary valve flow and tricuspid valve flow enables assessment of RV diastolic function in the presence of PR [26]. Altered flow hemodynamic forces have also been suggested as a feature of mechanical myocardial dyssynchrony in heart failure, cardiomyopathies, and TOF [32].

Other studies observed ventricular KE as a promising non-invasive early indicator of ventricular efficiency [31, 33, 34, 36, 37]. A correlation between pathologically turbulent flow and abnormal vascular remodeling [67] is consistent with greater energy loss in rTOF with increased ventricular remodeling [30, 36]. Turbulent kinetic energy patterns and anatomical location visualization in the RV in patients with more severe PR suggest that these reflect the severity of PR [31]. RV KE was also found to be higher in rTOF than healthy subjects [36] indicating that more work needs to be done by the RV in rTOF to generate the same cardiac output as in normal subjects [52]. Future studies are needed to determine the predictive power of KE measurements for RV dysfunction and thus the timing of reintervention [52].

### Pulse sequence parameter recommendations based on the systemic review outcome for rTOF patient scan

These recommendations are based on the results of the systematic review, in comparison with the 2015 SCMR 4D flow consensus statement [16]. Although parameters change according to the different clinical indications, future multi-center clinical studies would benefit from standardization of pulse sequence parameters.

### Respiratory and cardiac gating

Retrospective ECG synchronization had been recommended [16] to avoid overlooking data in the last 10–20% of the cardiac cycle. Prospective gating can be used when quantifying parameters such as peak systolic velocity or net flow which are low at the end of the cycle [21]. This was supported by high correlation between 2D PC-CMR and 4D flow CMR results. However, regurgitant fraction had lower Bland Altman agreement, supporting the recommendation of retrospective ECG gating. Respiratory gating on the other hand, although recommended [16], was not done by more than half the studies included. Free-breathing 4D flow CMR without respiratory gating decreases the scan time and in some cases had had negligible effect on accuracy [11]. However, future studies should endeavour to apply both retrospective ECG gating and some form of respiratory gating or motion correction.

### Contrast enhancement

4D flow utilizes bright blood spoiled gradient echo sequences, enabling the generation of PC angiograms without the need for an external contrast agent. This allows application to patients that are either contraindicated or are at risk [16]. More than half the included rTOF studies used gadolinium, often because it was required for other reasons. T1 shortening contrast agents enable larger flip angles and reduced signal loss due to saturation in 4D flow acquisitions, and are commonly available in centers [24]. This agrees with the consensus statement [16]; however, timing of administration and type used may cause variable outcomes.

### Velocity encoding (VENC)

Standard 4D flow acquisitions have required a single VENC adjusted to the highest expected velocity within the chosen 4D volume [16]. This prevents aliasing but poses a potential inaccuracy for quantification of low velocities in venous or intra-cardiac flows. For rTOF studies a VENC of 150 cm/s was a common compromise. Although some studies found an average error of less than 3% of measured flow volumes [10], VENC may need to be customized according to the needs of each case [30]. Dual VENC sequences have been proposed to avoid aliasing in fast velocities and retain high signal to noise ratio in low flow regions [51]. Recently, a triple VENC [68] method has been developed. The recommended VENC of 150 cm/s has been used by studies that evaluated vortices [12, 18]. However, most studies recorded a range and it was not always clear what VENC was used for what purpose. It is recommended that future studies report these details for ease of replication, with a minimum of one VENC set at 150 cm/s unless otherwise indicated.

### Temporal and special resolutions

It has been recommended that at least 5–6 voxels across the vessel lumen are essential for quantitative volume accuracy, and 2.5 × 2.5 × 2.5 mm^3^ was recommended for the aorta or pulmonary artery in the consensus statement [16]. However, the reviewed studies had a tendency to be lower (0.8 mm at lowest) probably to have better visualization of branch pulmonaries or veins. Also the echo time should be minimized to reduce signal loss due to incoherent flow patterns as shorter echo times have been suggested to further improve precision of flow measurements [27].

Temporal resolution < 40 ms [16] with a maximum voxel size of 2.5 × 2.5 × 2.5 mm^3^ is recommended, which was in agreement with most studies included. Higher temporal resolution imaging techniques were suggested to improve future studies that aim to further clarify fluid-tissue interaction relationships [35].

### Postprocessing corrections

Eddy-current correction and Maxwell correction techniques should be employed to reduce background phase-offsets [9, 29, 43]. Phase unwrapping also affects the accuracy of velocity measurements at peaks and should be employed. This is in agreement with the consensus statement [16] and was employed by all the studies reviewed.

### Software

A variety of software packages were used across studies; some in-house tools have been developed [28, 42] but more recent studies typically used commercially available packages [24, 25] that are either provided on the scanner or on post-processing workstations, utilizing the DICOM format. Consistency between vendors is highly recommended but currently not well characterized. Ideally, open benchmark datasets should be used to evaluate consistency between software solutions.

### Limitations

4D flow CMR is a rapidly evolving field, and current study designs have considerable methodological heterogeneity. Until recently, most software used in-house code which was not commercially available. Closed source analysis software makes it difficult to evaluate the accuracy of results or the details of methodology. Acquisition durations are still lengthy, and the use of contrast is common, although advances in machine learning show promise in enhancing 4D flow data [69, 70]. Almost half the number of studies had missing information that would impact the reproducibility of the work, e.g. the number of phases was not reported in 12/26 studies. There was also a lack of clinically relevant endpoints correlated with 4D flow. Future research is required to develop multicenter studies. The nature of systematic reviews only allows use of existing literature, and biases and limitations of included studies affects the reliability of the review.

## Conclusion

4D flow is highly applicable to rTOF patients in a single free-breathing 10–15 min acquisition. Particular strengths are retrospective valve tracking, velocity profiling, and volumetric quantification. Novel parameters such as KE and vortex quantification offer new insights into mechanisms of disease. Prospective, randomized, multi-centered studies are required to investigate the application of these methods in patient management.

## Data Availability

This research has been conducted using published studies.

## Abbreviations

2D: Two-dimensional
4D: Four-dimensional
bSSFP: Balanced steady state free precession
CASP: Critical Appraisal Skills Programme
CHD: Congenital heart disease
CMR: Cardiovascular magnetic resonance
ECG: Electrocardiogram
EDV: End-diastolic volume
ECG: Electrocardiogram
ESV: End-systolic volume
KE: Kinetic energy
LV: Left ventricle/left ventricular
PC: Phase contrast
PICS: Parallel imaging compressed sensing
PR: Pulmonary regurgitation
rTOF: Repaired tetralogy of Fallot
RV: Right ventricle/right ventricular
TOF: Tetralogy of Fallot
TR: Tricuspid regurgitation
VIPR: Isotropic voxel radial projection imaging
VENC: Velocity encoding
VSD: Ventricular septal defect
